# The “Texas Health College” Matter

**Published:** 1897-04

**Authors:** 


THE "TEXAS HEALTH COLLEGE" MATTER.—Replying to an inquiry by the president of one of the district boards of medical examiners in Texas, as to what course the Board should pursue in the case of a person engaged in practice by virtue, alone, of a "diploma" emanating from the so-called "Texas Health College," alleged to be situated at Mount, Coryell county (March number, page 498) it will be recalled that we said—"In view of the inaction of the attorney-general, notwithstanding his promise to us to interest himself in the matter, and also interest and instruct local attorneys, the gentleman from Coryell and others, say that you ought to have the parties arrested for practicing medicine in violation of the penal code."

The attorney-general takes exception to the above statement, and says it is unjust. That it is unintentionally so need not be said: we were not in possession of all the facts. The correspondence between us has elecited the following letter, which, as it is upon a matter of general and special interest to the medical profession, we publish herewith, much gratified to have the attorney-general's assurance of interest, and hearty co-operation in efforts to remedy the evil. We advise our readers to promptly notify the attorney-general of the use of these diplomas when a case comes to their knowledge.

ATTORNEY GENERAL'S OFFICE.
AUSTIN, TEXAS, March 20. 1897.

*Dr. F. E. Daniel, Austin, Texas:*

DEAR SIR:—I felt well assured that you had not intentionally done me injustice. In my letter I stated to you that I had "uniformily advised the district and county attorneys that it could not be doubted that the so-called college was a fraud," that these diplomas should not be recognized and that parties using them were to be dealt with the same as if they had no diploma at all, etc. I did not mean to convey the idea, nor do I now wish to be understood as saying that I have advised every district attorney in Texas, or every county attorney in Texas, on the point mentioned. But I have advised a number of them to that effect, and at all times wherever the question was raised. My correspondence on this subject has been considerable. I have endeavored to make it "general" in the sense of applying it to every county where the holders of these fraudulent diplomas are practicing. I, of course, had no idea that they were scattered over every county in the State, nor do I now understand such to to be the fact.

Your assumption that this office could deal with these matters collectively is an erroneous one. I have no authority to deal with it directly at all. Should I bring proceeding to forfeit the so-called charter of that institution, a judgment to that effect would not interfere with diplomas already granted. I can institute no prosecutions in cases of that character (to compel to forfeit charters). The constitution and statutes of the State put the control of such cases in the trial courts in the hands of the district and county attorneys.

I felt sure your article was written upon a misapprehension, both as to what has been done and as to what the law authorizes this department to do.

Very truly yours, M. M. CRANE.

